# Filtration of Active Components with Antioxidant Activity Based on the Differing Antioxidant Abilities of *Schisandrae Sphenantherae Fructus* and *Schisandrae Chinensis Fructus* through UPLC/MS Coupling with Network Pharmacology

**DOI:** 10.1155/2021/5547976

**Published:** 2021-07-21

**Authors:** Yang Xin, Yang Yang, Kaichen Yu, Haijun Wang

**Affiliations:** ^1^College of Chemistry and Chemical Engineering, Qiqihar University, Qiqihar 161006, China; ^2^Heilongjiang Academy of Chinese Medical Sciences, Harbin 150036, China; ^3^Heilongjiang Provincial Key Laboratory of Catalytic Synthesis for Fine Chemicals, Qiqihar University, Qiqihar 161006, China; ^4^School of General Medicine and Continuing Education, Qiqihar Medical University, No. 333, Bukui Street, Qiqihar 161006, China

## Abstract

This study attempted to filter active components with antioxidant activities based on the differing antioxidant abilities of *Schisandrae Sphenantherae Fructus* (*SSF*) and *Schisandrae Chinensis Fructus* (*SCF*). First, the antioxidant activity of *SSF* and *SCF* was evaluated through the DPPH free radical scavenging method and compared with the half maximal inhibitory concentration (IC_50_) value. Next, components of *SSF* and *SCF* were detected by employing ultrahigh-performance liquid chromatography-Q-Exactive Orbitrap mass spectrometry (UPLC-QEO/MS) technology, and differential compounds were screened out as potential antioxidant compounds by using Compound Discover 3.1 Software. After that step, in order to verify the antioxidant compounds, the network method was applied. Biological targets were searched in the GeneCards database, and that related to antioxidant ability were selected in the Comparative Toxicogenomics Database (CTD). Finally, the pharmacology network was constructed. Results showed that *SSF* and *SCF* possessed different compounds and antioxidant abilities. A total of 14 differential compounds such as *γ*-schizandrin, schisandrin B, schisandrin, and tigloylgomisin H between them were screened out and identified. Twenty targets associated with antioxidant activity contained MAP2K1, MAPK8, RPS6KB1, PRKCB, HIF1A, and so on were investigated. Thirty-six pathways contained HIF-1 signaling pathways, choline metabolism in cancer, serotonergic synapse, Fc epsilon RI signaling pathway, GnRH signaling pathway, and so on related to the above twenty targets were identified. The pharmacology network analysis indicated that the differential components may be helpful in treating various diseases, especially cancer, by exerting antioxidant activity. In conclusion, this study provided a novel method for identifying active components with antioxidant activity in *SSF* and *SCF*, and this method may be applicable for the filtration of bioactive components in other herbs.

## 1. Introduction

Antioxidation is a process that can effectively inhibit the oxidation reaction caused by free radicals. The mechanism of antioxidation involves either an action on free radicals or the consumption of substances that can easily generate free radicals.

Free radicals are constantly produced in the body due to exposure to the environment through processes such as respiration (oxidation reaction), air pollution, and radiation. Free hydroxyl radicals are associated with aging stress [1], autoimmunity [2], bone loss, and cancer development [3]. However, substances that possess antioxidant activity can terminate or inhibit the oxidation process by scavenging free radicals [4]. Thus, maintaining the balance of free radicals and the antioxidant active substance in the body can delay aging. Therefore, finding antioxidant active components is of significance for developing antioxidant health products and pharmaceuticals to cure diseases caused by oxidative damage, as well as for studying the mechanisms governing antioxidant activity.

*Schisandrae Sphenantherae Fructus* (*SSF*) and *Schisandrae Chinensis Fructus* (*SCF*) are both well-known Chinese herbal medicines, and *SSF* is primarily produced in the central and southern part of China, whereas *SCF* is primarily produced in northern China. Although these medicines possess some similar pharmacological actions, such as antioxidant, anti-inflammatory, and anxiolytic effects [5], *SCF* and *SSF* exhibit differences in chemical components [6] and other pharmacological actions [7], which might be the reason why *SCF* is more popular in Chinese medicine than *SSF*. Therefore, conducting a study simultaneously comparing the components and biological activities, as well as their connections, was of interest.

Regarding Chinese herbal medicine, because of the multiple components of these medicines, the interactions between them and treated organisms is very complex, which makes studies on the mechanisms governing the medicines difficult to conduct. Fortunately, the concept of “network pharmacology” was proposed by Hopkins in 2008 [8] and has been widely employed in research on Chinese herbal medicine [9]. With the help of network pharmacology, the correlation between drugs and action targets could be predicted through computer virtual computation, network database retrieval, network modeling, and network analysis. Compared to traditional pharmacology, network pharmacology exhibits the features of “multitarget, multieffect, and complex disease” [10]. Based on the above characteristics, network pharmacology has been recognized by an increasing number of scholars, and network pharmacology has been increasingly utilized to study pharmacological effects.

In this study, a network pharmacology method was employed to verify the antioxidant active compounds filtered by comparing the components in *SCF* with those of *SSF* based on the results of UPLC/MS technology. Specifically, this report presents a novel method for determining the antioxidant components of these medicines, which depends on connecting the differing antioxidant abilities of *SCF* and *SSF* with the differential components between them, as well as verifying these results through the strategy of network pharmacology. The method described in this study may facilitate the identification of antioxidant compounds in other herbal medicines.

## 2. Experiment

### 2.1. Solvent and Medicine

*Schisandrae Chinensis Fructus* (*Schisandra chinensis* (Turcz.) Baill., fruit, dried) was purchased from Qi Tai Pharmacy (Qiqihar, China). *Schisandrae Sphenantherae Fructus* (*Schisandra sphenanthera* Rehd. et Wils., fruit, dried) was purchased from Xing Kang Pharmacy (Qiqihar, China). The reference standard for 1,1-diphenyl-2-picrylhydrazyl (DPPH) was purchased from Avanti (Alabama, USA), and L-ascorbic acid (vitamin C (VC)) was purchased from InnoCHEM (InnoCHEM, China). Analytical-grade anhydrous ethanol was used (Tianli, China).

### 2.2. Sample Preparation

*SCF* and *SSF* were prepared using the reflux extraction process [11]; the extraction method for them was optimized based on the DPPH radical clearance rate by utilizing an L9(3^4^) orthogonal design. During orthogonal experiments, factors concerning extract times, reflux time, and material-liquid ratio were all inspected with four levels. The optimal extraction process was as follows: first, *SCF* or *SSF* was weighed 2.5 g. Next, the herbal medicine was mixed with 20 volumes of water and then boiled for 2 h, 5 times. The extract was then filtered, mixed, and concentrated to 50 mL. After that, 2 mL of the extract was measured and dried. Finally, the residue was dissolved using 2 mL anhydrous ethanol before detection.

### 2.3. DPPH Radical Clearance Test

DPPH was dissolved with 95% ethanol aqueous solution to prepare 0.1 mmol/L of DPPH solution and 2 mL of it was mixed with 2 mL sample. The mixture was then detected at 517 nm using a UV spectrometer after 30 min.

### 2.4. Condition of Detection

The absorbance of the *SCF* and *SSF* extracts was measured on an ultraviolet-visible spectrophotometer TU1901 (Persee Co., China) in the spectral scan mode in the range of 510∼516 nm.

Compounds in *SCF* and *SSF* were detected on UPLC-MS equipment consisting of a Dionex UltiMate 3000 UHPLC and Q-Exactive Orbitrap MS with an electrospray source in positive-ion mode (Thermo Co., USA).

UPLC separation was performed on a TM C18 column (2.1 × 50 mm, 2.6 *μ*m, Thermo Co., USA). Formic acid was added to the mobile phase, which consisted of methanol (A) and 0.1% formate water (B), to improve ionization efficacy and acquire a better peak shape. The flow rate was set at 0.3 mL·min^−1^. UPLC resolution was optimized with gradient elution as follows: 0∼2 min, 40%∼60% A; 2∼6 min, 60%∼100% A; and 6∼8 min, 100% A. The column temperature was 25°C, and the injection volume was 0.5 *µ*L.

The optimal mass spectrometry signal was obtained as follows: capillary (+4.0 kV), desolvation temperature (350°C), S-lens voltage (50 V), shield gas (35 arb), aux gas (10 arb), scan type (full scan), and scan range (100∼1200 Da). The collision energy for MS^2^ was set at 10, 30, and 40 eV to acquire abundant fragment ions.

The UV method was confirmed by inspecting the accuracy and stability of the absorbance of the same *SCF* sample six times. The stability was determined by testing six times in 30 min. A relative standard deviation percentage (RSD%) of absorbance below 1% was considered to indicate a good detection method.

The UPLC-MS method was validated by inspecting the accuracy and stability through the detection of six compounds in both *SCF* and *SSF*. The accuracy was determined by injecting the same *SCF* sample continuously six times. The stability was implemented by injecting the same *SCF* sample at 0, 12, 24, 32, 40, and 48 h. The RSD% of retention time and peak area below 5% was considered to indicate a good detection method.

### 2.5. Data Analysis

The UV absorbance data of *SSF* and *SCF* were used to calculate the DPPH radical clearance, as well as the IC_50_ value. First, the DPPH radical clearance was calculated as (Abs_blank_−Abs_sample_)/Abs_blank_ × 100%. Second, the IC_50_ value was acquired through curve fitting, with extract concentrations of 0.2, 0.4, 0.6, 0.8, and 1.0 mg/mL serving as the *X*-axis and the DPPH radical clearance value serving as the *Y*-axis, by GraphPad software. Third, the IC_50_ values of the medicines were compared with that of vitamin C to estimate their antioxidant ability.

All UPLC/MS raw data were acquired by Q-Exactive Tune (Thermo Co., USA) and processed with Compound Discoverer 3.1 (Thermo Co., USA). Next, a data list containing data such as molecular weight, retention time, peak intensity, and *p*

value of *SCF* versus *SSF* was acquired. Compounds with a *p*

value <0.05 were considered to be significantly different between *SCF* and *SSF*. Principal component analysis (PCA) was used to observe the overall classification of the data. Next, the differential compounds were identified by searching the mzCloud, ChemSpider, PubChem, NIST Chemistry WebBook databases, and references based on their MS^2^ spectra.

The targets of differential compounds were screened out in the Swiss Target Prediction database after the canonical SMILES number was obtained from PubChem and compounds were standardized by the UniProt database. The CTD and GeneCards databases were searched for the targets of antioxidant function. After that step, the common targets of differential compounds and antioxidant function were imported into the STRING database, and “multiple proteins” and “human sources” were checked. Next, protein-protein interactions (PPIs) were depicted using Cytoscape. Next, Gene Ontology (GO) and Encyclopedia of Genes and Genomes (KEGG) enrichment analyses were performed using the Metascape database. Finally, a network pharmacology diagram was drawn with the help of Cytoscape software.

## 3. Results

### 3.1. Evaluation of Methodology

Both of the methodologies for UV and UPLC/MS were performed. For UV, the RSD% of accuracy and stability was less than 0.5%. Regarding the UPLC/MS method, the RSD% accuracies for schisandrin, gomisin J, schisantherin B, angeloylgomisin P, schilancifolignan A, and schisantherin C were 0.97%, 1.64%, 1.84%, 1.72%, 0.85%, and 2.04%, respectively, and the RSD% of stability for them in *SSF* was 2.55%, 2.44%, 2.90%, 0.68%, 2.98%, and 3.52%, respectively, whereas those in *SCF* were 3.36%, 3.60%, 0.45%, 3.24%, 4.95%, and 0.91%, respectively. All the RSD% values were less than 5%, which indicated that the accuracy and stability of the analytical method were acceptable.

### 3.2. Filtration of the Optimal Extraction Process for Antioxidant Activity

The results of the orthogonal test are shown in Table 1. It can be seen that, through the optimization of the extract method, sample prepared by the extraction process with the material-liquid ratio of 1 : 20, extracted for 5 times and 2.0 h/time showed the optimal DPPH radical clearance rate, which is used for the extraction of *SSF* and *SCF*.

### 3.3. Estimation of Antioxidant Activity between *SSF* and *SCF*

The DPPH radical clearance ability of *SSF* and *SCF* was estimated by comparing the IC_50_ value with vitamin C (VC); the lower the IC_50_ value was, the better the antioxidant activity was. The results showed that the IC_50_ values of *SCF*, *SSF*, and VC were 0.4518 ± 0.0008 mg/mL, 0.4928 ± 0.0015 mg/mL, and 0.2225 ± 0.0037 mg/mL, respectively, which indicated that *SCF* exhibited better antioxidant ability than *SSF*.

### 3.4. Filteration and Identification of the Potential Antioxidant Active Components

To determine the reason for the antioxidant ability of *SSF* being better than that of *SCF*, it was surmised that there were some compounds that showed a higher intensity in *SCF* than in *SSF*; therefore, the differential compounds between *SSF* and *SCF* were filtered.

**Figure 1:**
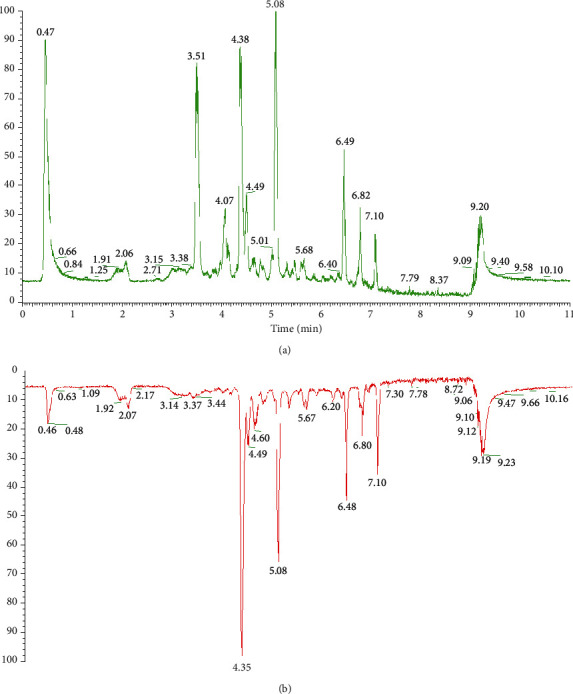
Mirror image of the total ion chromatogram for *SCF* (a) and *SSF* (b).

First, samples of *SSF* and *SCF* were detected by UPLC/MS, and total chromatograms were obtained using Q-Exactive Tune (Thermo Co.). The clear difference in the peak amount and intensity between *SSF* and *SCF* is depicted in Figure 1.

**Figure 2:**
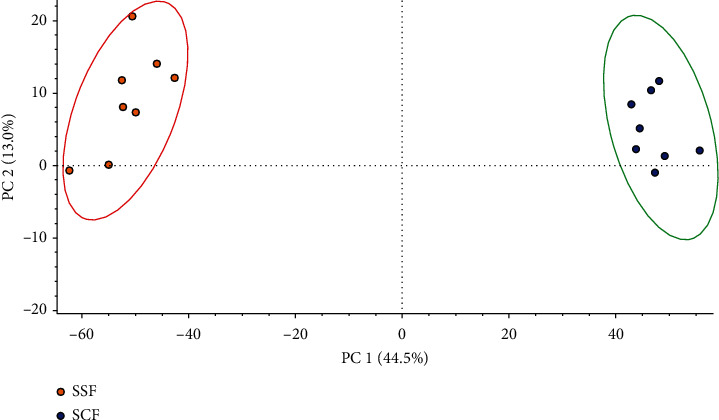
PCA for samples of *SCF* and *SSF*.

Second, the overall difference between *SSF* and *SCF* was observed through unsupervised principal component analysis (PCA) (Figure 2), and variables making significant contributions to discrimination between them were selected based on a *p* value <0.05 by using Compound Discoverer 3.1 software. Next, the Traditional Chinese Medicine Systems Pharmacology Database (TCMSP, https://tcmspw.com/tcmsp.php), mzCloud (https://www.mzcloud.org/), ChemSpider (https://www.chemspider.com/), PubChem (https://pubchem.ncbi.nlm.nih.gov/), and the National Institute of Standards and Technology (NIST) Chemistry WebBook (https://webbook.nist.gov/chemistry/) were searched to determine the molecular weight and the molecular formula of different variables, and their MS^2^ spectra were compared with those in references to identify the chemical structures. A total of 14 compounds were identified, and all their intensity ratios in *SCF* versus *SSF* were greater than 1; therefore, they were initially considered antioxidant compounds, as shown in Table 2.

**Figure 3:**
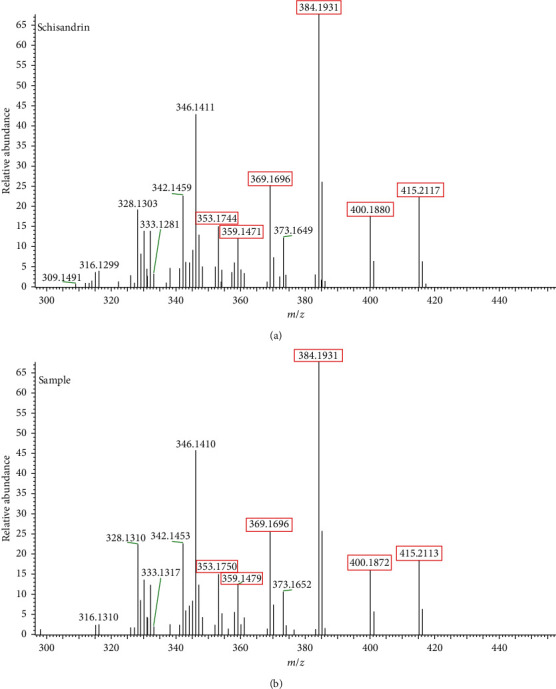
The MS^2^ spectrum of schisandrin in the standard and sample solution.

**Figure 4:**
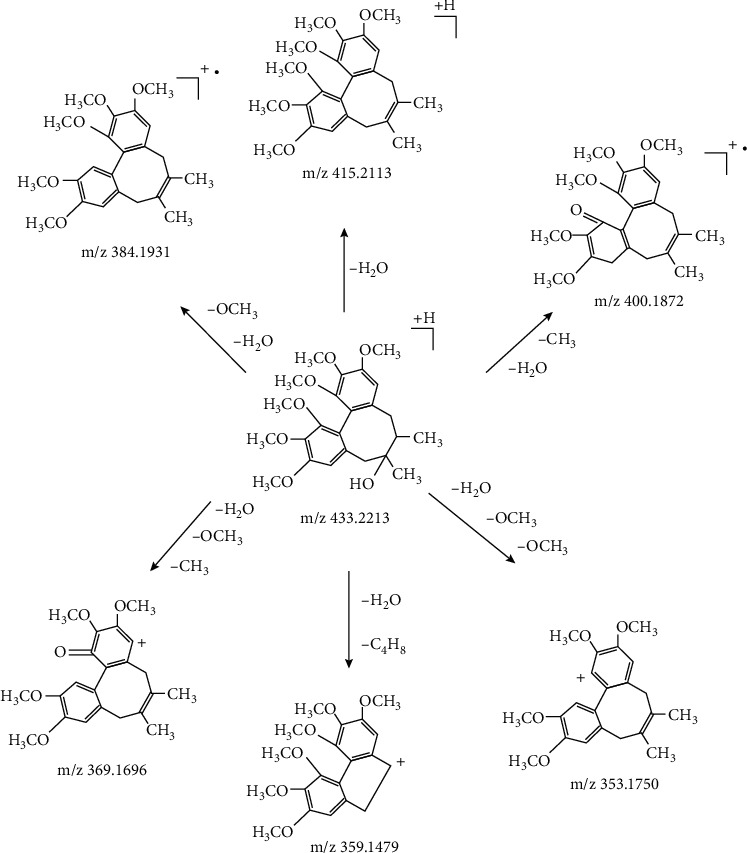
The proposed fragmentation pathways of schisandrin.

Among these compounds, espatulenol was proposed by comparing the MS^2^ spectrum with that in the NIST Chemistry WebBook database, *γ*-schizandrin, schisantherin B, schisantherin C, tigloylgomisin H, gomisin J, angeloylgomisin P, angeloylgomisin H, gomisin M1, gomisin E, and gomisin K1 were proposed by comparing the MS^2^ spectrum with that in references [12–17], and schilancifolignan A was speculated based on its MS^2^ fragments [18]. Schisandrin and schisandrin B were verified by their standards. The ion of *m*/*z* 455.2040, which was one significantly different compound between *SSF* and *SCF*, was selected as an example to illustrate the compound identification process. First, the extract ion spectrum of *m*/*z* 455.2040 was acquired at *t*_R_ 3.71 min. Then, its MS^2^ spectrum was acquired under 40 eV, which is shown in Figure 3. Figure 3 shows that fragment ions of *m*/*z* 415.2113, 400.1872, 384.1931, 369.1696, 359.1479, and 353.1750 exerted the same fragmentation with that of schisandrin reported in references, corresponded to [M + H-H_2_O]^+^, [M + H-H_2_O-CH_3_]^+^, [M + H-H_2_O-OCH_3_]^+^, [M + H-H_2_O-OCH_3_-CH_3_]^+^, [M + H-H_2_O-C_4_H_8_]^+^, and [M + H-H_2_O-OCH_3_-OCH_3_]^+^ based on high-resolution mass spectrometry. Hence, it was deduced to schisandrin initially. Further, the schisandrin standard was detected by the same UPLC/MS condition for verifying the deduction, which showed that their retention time and MS^2^ spectrum were matched completely. Therefore, the ion of 455.2040 was identified as schisandrin. The proposed fragmentation pathways of schisandrin are shown in Figure 4.

### 3.5. Prediction of the Targets of Antioxidant Compounds

A total of 441 targets on which the abovementioned potential antioxidant compounds acted were obtained. At the same time, 4151 targets associated with antioxidant activity were obtained by searching the GeneCards database, and 3946 of them were retained after UniProt processing. Furthermore, 4531 targets related to antioxidant activity were obtained by searching the CTD database, and 150 of them were retained after UniProt processing. There were 147 common targets in the GeneCards database and CTD database. Furthermore, the 147 targets were compared to the abovementioned 441 targets, and it was found that 20 targets on which the antioxidant compounds acted were related to antioxidant activity; these targets are listed in Table 3.

### 3.6. Protein-Protein Network Construction

**Figure 5:**
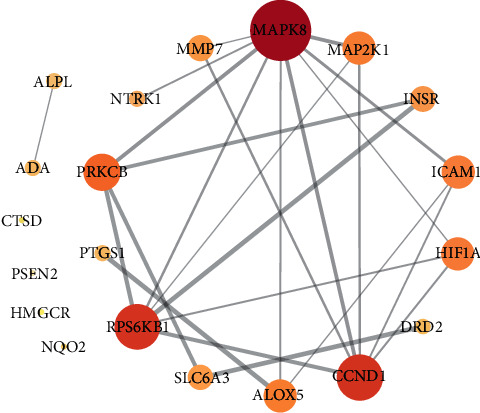
Protein-protein interaction network.

The protein-protein interaction (PPI) network diagram was constructed using Cytoscape software (Figure 5). Figure 5 shows that the network graph contained targets and 24 edges. Each target connected several functions. The larger the degree value was, the larger the node was, and the larger the combined score value was, the thicker the edge was. Among these genes, the top 3 genes with larger nodes included MAPK8, CCND1, and RPS6KB1, which indicated that they were important targets on which the potential antioxidant components acted.

### 3.7. Gene Ontology Enrichment Analysis for Targets

**Figure 6:**
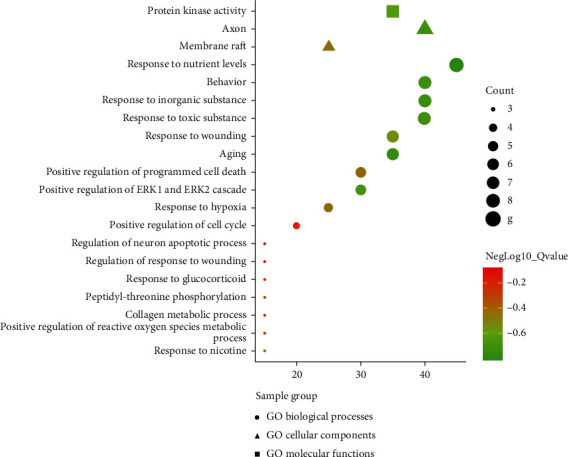
Target biological enrichment analysis.

**Figure 7:**
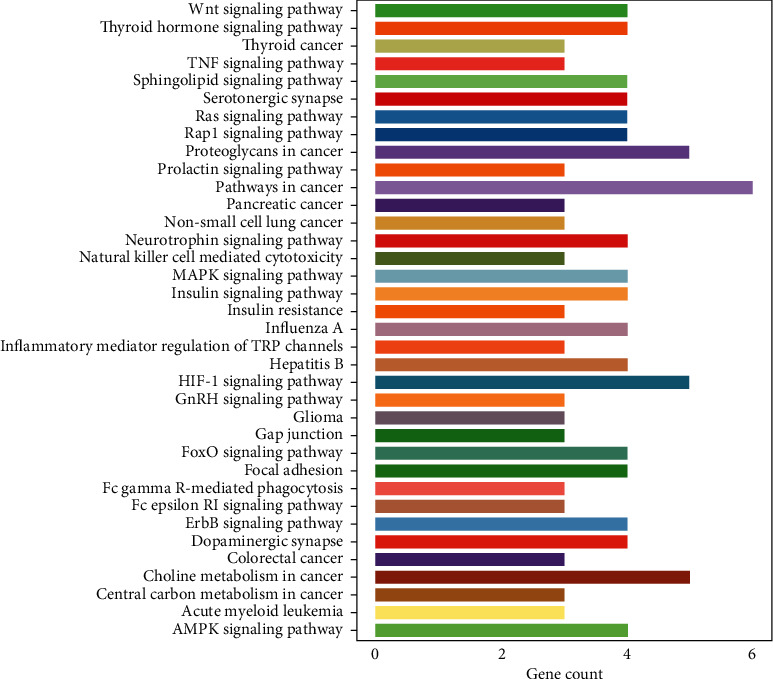
KEGG enrichment pathway analysis.

GO (gene ontology) enrichment analysis of potential targets was performed using the Metascape database, and the functions were enriched under the conditions of *p*

value <0.01 and enrichment factor >1.5.  Figure 6 shows the top 20 most significant functions. In Figure 7, the point size represents the number of genes with the same function; the larger the point size is, the more genes there are. The results showed that potential antioxidant compounds affected numerous biological functions involving biological processes, cellular components, and molecular functions, such as the regulation of aging, the active regulation of programmed cell death, the positive regulation of reactive oxygen metabolism, the regulation of neuronal apoptosis, positive regulation of the cell cycle, and regulation of the collagen metabolism process. These targets might slow the occurrence and development of aging and hypoxia caused by the oxidation process by participating in the process of antioxidant reactions.

### 3.8. Pathway Analysis for Targets

The top 36 enriched pathways of the above 20 targets were obtained from the DAVID (Database for Annotation, Visualization, and Integrated Discovery) database, which is shown in Figure 7. The length of the column represents the number of genes participating in the pathway; the longer the column is, the more genes there are. Among these pathways, pathways in cancer, the HIF-1 signaling pathway, insulin resistance, the insulin signaling pathway, hepatitis B, the AMPK signaling pathway, the MAPK signaling pathway, the TNF signaling pathway, and the FoxO signaling pathway were reported to be related to antioxidant activity [19–27].

### 3.9. Construction of Compound-Target-Pathway Network

**Figure 8:**
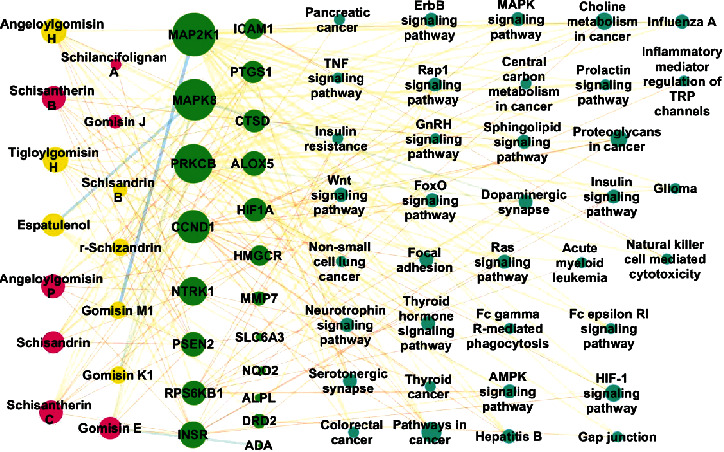
Compound-target-pathway network.

The compound-target-pathway network was constructed by connecting 14 differential compounds, 20 targets, and 36 pathways.  Figure 8 shows that there were a total of 70 nodes and 230 edges. The larger the node was, the more nodes were connected to it. Therefore, angeloylgomisin H, aigloylgomisin H, angeloylgomisin P, schisantherin C, and schisantherin B were deemed to have stronger antioxidant activity than other compounds. MAP2K1, MAPK8, PRKCB, and CCND1 were considered to be the primary antioxidant effect targets. Pathways in cancer, choline metabolism in cancer, and proteoglycans were considered to be the primary pathways involved in antioxidant processes.

## 4. Discussion

*SSF* and *SCF*, which serve as typical “medicine-food homology” herbal medicines, showed similar pharmacological effects, such as antioxidant activity. Nevertheless, *SCF* was generally more popular than *SSF* because it was said that *SCF* possessed higher quality than *SSF*. Regarding *SSF* and *SCF*, because both of these medicines had multipharmacological effects, the main effects that were essential for quality differences were not clear. The antioxidant activity or components of medicines have long been compared by medicinal scholars [28, 29], but to the best of our knowledge, studies on the relationship of antioxidant ability and differential components have not been performed to date. Therefore, this study described their relationship and predicted the functional targets of antioxidant activities using a network pharmacology strategy. The compound-target-pathway network diagram is shown in Figure 8, in which there were a total of 70 nodes and 230 edges. The larger the degree value was, the larger the nodes were, and the stronger the antioxidant activity of the compounds was. Among the 14 differential compounds, seven compounds, namely, *γ*-schizandrin, espatulenol, angeloylgomisin H, tigloylgomisin H, schisantherin B, gomisin K1, and gomisin M1, which were colored yellow, were unique components in *SCF* that were not observed in *SSF*. In addition, five compounds, namely, angeloylgomisin H, tigloylgomisin H, angeloylgomisin P, schisantherin C, and schisantherin, showed larger nodes than others, which indicated that they were the main antioxidant components. Among the 20 targets, four, namely, MAP2K1, MAPK8, PRKCB, and CCND1, showed larger nodes than the others, which indicated that they were the main targets of antioxidant functions. Among the 36 pathways, pathways in cancer, choline metabolism in cancer, and proteoglycans in cancer showed larger nodes than others, which indicated that they were the main pathways through which the abovementioned bioactive compounds exerted antioxidant function.

As a dual-specific kinase, mitogen-activated protein kinase kinase 1 (MAP2K1) participates in the extracellular regulated protein kinase (ERK) pathway by activating ERK1 and ERK2. Through the identification of transforming genes for refractory diseases in the sclerosing subtypes of highly invasive tumors of gastric cancer, it was determined that cancer cells are dependent on the increased proliferation activity of MAP2K1, and MAP2K1 could be utilized as a cancer inhibitor target [30].

MAPK8 (mitogen-activated protein kinase 8), which is a protein-coding gene, was observed to participate in the abovementioned 16 pathways and to connect to 9 targets in the PPI network diagram, which indicated its importance. MAPK8-associated diseases include fatty liver disease and hepatitis C. The pathways related to MAPK8 include the ATM (serine/threonine kinase) pathway and the link between physicochemical characteristics and toxicity-related pathways. Previous studies showed that MAPK8 is involved in the gene expression process of anterior papillary hypertension caused by hypoxia [31].

PRKCB (protein kinase C) has been identified as a drug treatment target for specific diseases involved in various cellular processes, such as the regulation of B-cell receptor (BCR) signaling bodies, oxidative stress-induced apoptosis, male hormone receptor-dependent transcriptional regulation, insulin signaling, and endothelial cell proliferation. PRKCB, as the locus of systemic lupus erythematosus [32], was observed to have upregulated mRNA expression in the peripheral blood mononuclear cells of patients with systemic lupus erythematosus.

MAPK8 and HIF1A were important targets in choline metabolism. Abnormal choline metabolism may serve as an indicator of tumorigenesis and tumor progression. Increased levels of MAPK8 and HIF1A could induce the expression of related genes and subsequently affect the cell growth and proliferation [33]. Therefore, it could be deduced that the differential compounds might help to treat cancer by acting on MAPK8 and HIF1A.

As a cyclin, CCND1 participates in a variety of gene expression and protein pathways. CCND1-associated chromosomal aberrations could cause multiple occurrences of lymphocyte malignant tumors, rack mounting, and multiple occurrences. CCND1 and its catalytic partner cyclin-dependent kinase 4 (cdk4) play important roles in the G1/S checkpoint of the cell cycle. Takano et al. [34] hypothesized that the synergistic effect of CCND1 and cdk4 with ER may cause breast cancer.

Through pathway analysis, it was observed that the antioxidant activities of *SSF* and *SCF* were closely related to the HIF-1 (hypoxia-inducible factor 1) signaling pathway, pathways in cancer, and choline metabolism in cancer. HIF-1 is a transcription factor and a major regulator of oxygen homeostasis. This protein consists of an inducible HIF-1-alpha subunit and a constitutively expressed HIF-1beta subunit. Under normoxia and hypoxia, the conversion of the two subunits regulates their transcriptional activity. Under hypoxia, HIF-1 is the main regulator of many hypoxia-induced genes. HIF-1-related factors may cause such diseases as diabetic retinopathy, glucocorticoid-induced osteonecrosis, and malignant paraganglioma [35].

Antioxidants were frequently used for cancer treatment in previous studies [36], and angeloylgomisin H was reported to be cytotoxic to human cancer cell lines [37], which suggested that antioxidant active compounds might also possess anticancer activity. Among the 36 pathways obtained in this study, 9 related to pathways in cancer accounted for 25%, which indicated that *SCF* and *SSF* might serve as cancer treatments by exerting antioxidant activity.

## 5. Conclusion

In this study, the antioxidant activity of *SSF* and *SCF* was assessed and compared by DPPH free radical scavenging experiments, and the differential compounds, as well as their associated biological functions, were obtained by coupling UPLC/Q-Exactive Orbitrap MS technology with network pharmacological analysis. The results showed that the antioxidant ability of *SCF* was stronger than that of *SSF*, and 14 differential compounds were identified between *SCF* and *SSF*, which indicated that these compounds were potential active components with antioxidant activity. Further analysis showed that there were a total of 20 predicted targets and 36 pathways related to these active components with antioxidant activity, and most of these targets and pathways were associated with cancer regulation. The pharmacology network predicted that the differential components might exert antioxidant effects on the 20 targets by regulating the 36 pathways. These medicines may be helpful for treating various diseases, especially cancer, by exerting antioxidant activity. Therefore, this study provided a novel method for identifying active components with antioxidant activity, and this technique may be applicable for the filtration of bioactive components in other herbs.

## Figures and Tables

**Table 1:** L9(3^4^) orthogonal array design matrix and experimental results.

No.	A	B (g/mL)	C (/h)	D	DPPH clearance%
1	3 (1)	1 : 08 (1)	1.5 (1)	1	22.81
2	3 (1)	1 : 10 (2)	2.0 (2)	2	27.34
3	3 (1)	1 : 20 (3)	2.5 (3)	3	27.50
4	4 (2)	1 : 08 (1)	2.0 (2)	3	26.88
5	4 (2)	1 : 10 (2)	2.5 (3)	1	26.72
6	4 (2)	1 : 20 (3)	1.5 (1)	2	30.63
7	5 (3)	1 : 08 (1)	2.5 (3)	2	30.47
8	5 (3)	1 : 10 (2)	1.5 (1)	3	30.16
9	5 (3)	1 : 20 (3)	2.0 (2)	1	41.39
K1	25.883	26.720	27.867	30.307	—
K2	28.077	28.073	31.870	29.480	—
K3	34.007	33.173	28.230	28.180	—
R	8.124	6.453	4.003	2.127	—

Note: A: reflow times; B: material-liquid ratio; C: reflow time; D: blank.

**Table 2:** Components of potential antioxidant activities.

No.	Deduced compound	*t*_R_ (min)	Elemental composition	Ion adduction	Calculated mass	Deviation (ppm)	MS^2^
1	*γ*-Schizandrin^*b,d*^	2.76	C_23_H_28_O_6_	[M + H]^+^	401.1959	−0.5	386.1723, 370.1768, 359.1487, 355.1532, 337.1432
2	Espatulenol^*b,d*^	3.40	C_15_H_24_O	[M + H]^+^	221.1900	−1.4	203.1795, 193.1587, 175.1481, 163.1481, 147.1168, 133.1012, 107.0858, 95.086
3	Schisandrin^*a,b*^	3.71	C_24_H_32_O_7_	[M + Na]^+^	455.2040	−2.0	455.2032, 415.2113, 400.1872, 384.1931, 369.1696, 359.1479, 353.1750
4	Tigloylgomisin H^*b,d*^	3.91	C_28_H_36_O_8_	[M + Na]^+^	523.2302	−0.6	493.1786, 455.2077, 423.1418, 401.1621, 383.1506
5	Gomisin J^*b*^	3.99	C_22_H_28_O_6_	[M + Na]^+^	411.1778	−1.5	357.1721, 325.1453, 319.1181, 297.1483, 287.0919
				[M + H]+	389.1943	−3.9	
6	Schisantherin B^*b*^ or angeloylgomisin P^*b*^	4.09	C_28_H_34_O_9_	[M + Na]^+^	537.2095	−0.7	437.1573,415.1750,371.1498,356.1246
7	Angeloylgomisin P^*b*^ or schisantherin B^*b*^	4.12	C_28_H_34_O_9_	[M + H]^+^	537.2095	−1.5	437.1573,415.1750,371.1498,356.1246
8	Angeloylgomisin H^*b,d*^	4.13	C_28_H_36_O_8_	[M + Na]^+^	523.2302	−0.6	416.1823, 387.1798, 372.1564, 356.1614, 342.1453, 326.1503
9	Schilancifolignan A^*c,d*^	4.25	C_24_H_30_O_7_	[M + H]^+^	431.2064	−1.2	493.1840,455.2039,423.1422,383.1489
10	Schisantherin C^*b*^	4.50	C_28_H_34_O_9_	[M + H]^+^	515.2275	−1.4	385.1649, 355.1543, 343.1183, 316.0944
11	Gomisin K1^*b,d*^	4.65	C_23_H_30_O_6_	[M + Na]^+^	425.1934	−0.7	410.1701, 395.1464, 379.1516
12	Gomisin E^*b*^	4.97	C_28_H_34_O_9_	[M + H]^+^	515.2275	−0.2	469.2206, 385.1646, 355.1538, 343.1179, 316.0941
13	Gomisin M1^*b,d*^	5.07	C_22_H_26_O_6_	[M + Na]^+^	409.1621	−1.0	394.1393, 363.1185, 333.1724
14	Schisandrin B^*a,b,d*^	5.37	C_23_H_28_O_6_	[M + H]^+^	401.1959	−0.7	300.0990,270.0883,242.0936,331.1174,386.1723,401.1953

The superscript “*a*” represents compounds identified by standard, superscript “*b*” represents compounds identified by literature reports, superscript “*c*” represents compounds speculated based on its MS^2^ fragments, and superscript “*d*” represents compounds solely found in *SCF*.

**Table 3:** Targets of potential antioxidant active compounds.

No.	UniProt ID	Gene name	Protein
1	P07339	CTSD	Cathepsin D
2	Q16665	HIF1A	Hypoxia-inducible factor 1 alpha
3	P06213	INSR	Insulin receptor
4	P45983	MAPK8	c-Jun N-terminal kinase 1
5	P04629	NTRK1	Nerve growth factor receptor Trk-A
6	P05362	ICAM1	Intercellular adhesion molecule 1
7	P04035	HMGCR	HMG-CoA reductase (by homology)
8	P05771	PRKCB	Protein kinase C beta
9	Q02750	MAP2K1	Dual specificity mitogen-activated protein kinase kinase 1
10	P23219	PTGS1	Cyclooxygenase-1
11	Q01959	SLC6A3	Dopamine transporter (by homology)
12	P09917	ALOX5	Arachidonate 5-lipoxygenase
13	P16083	NQO2	Quinone reductase 2
14	P14416	DRD2	Dopamine D2 receptor (by homology)
15	P00813	ADA	Adenosine deaminase
16	P23443	RPS6KB1	Ribosomal protein S6 kinase 1
17	P09237	MMP7	Matrix metalloproteinase 7
18	P05186	ALPL	Alkaline phosphatase, tissue-nonspecific isozyme
19	P24385	CCND1	Cyclin-dependent kinase 4/cyclin D1
20	P49810	PSEN2	Gamma-secretase

## Data Availability

The data used to support the findings of this study are available from the corresponding author upon request.

## Abbreviations

*SSF*: *Schisandrae Sphenantherae Fructus*
*SCF*: *Schisandrae Chinensis Fructus*
DPPH: 1,1-Diphenyl-2-picrylhydrazyl
VC: L-Ascorbic acid
IC_50_: Half maximal inhibitory concentration
UPLC/QEO MS: Ultrahigh-performance liquid chromatography-Q-Exactive Orbitrap mass spectrometry
CTD: Comparative Toxicogenomics Database
CTSD: Cathepsin D
HIF1A: Hypoxia-inducible factor 1-alpha
INSR: Insulin receptor
MAPK8: Mitogen-activated protein kinase 8
NTRK1: High-affinity nerve growth factor receptor
ICAM1: Intercellular adhesion molecule 1
HMGCR: 3-Hydroxy-3-methylglutaryl-coenzyme A reductase
PRKCB: Protein kinase C beta type
MAP2K1: Mitogen-activated protein kinase kinase 1
PTGS1: Prostaglandin G/H synthase 1
SLC6A3: Sodium-dependent dopamine transporter
ALOX5: Arachidonate 5-lipoxygenase
NQO2: Quinone reductase 2
DRD2: Dopamine receptor D2
ADA: Adenosine deaminase
RPS6KB1: Ribosomal protein S6 kinase beta-1
MMP7: Matrix metalloproteinase 7
ALPL: Alkaline phosphatase, tissue-nonspecific isozyme
CCND1: Cyclin-dependent kinase 4/cyclin D1
PSEN2: Presenilin-2
RSD: Relative standard deviation percentage
UV: Ultraviolet-visible spectrophotometer
UPLC-MS: Ultra high-performance liquid chromatography-mass spectrum in series
PCA: Principal component analysis
NIST Chemistry WebBook: National Institute of Standards and Technology (NIST) Chemistry WebBook
PPI: Protein-protein interaction
GO: Gene Ontology
KEGG: Kyoto Encyclopedia of Genes and Genomes
DAVID: Database for Annotation, Visualization, and Integrated Discovery
AMPK: AMP-activated protein kinase
MAPK: Mitogen-activated protein kinase;
TNF: Tumor necrosis factor
FoxO: Forkhead box O
ERK: Extracellular regulated protein kinase
ERK1: Extracellular regulated protein kinase 1
ERK2: Extracellular regulated protein kinase 2
TCMSP: Traditional Chinese Medicine Systems Pharmacology Database
ATM: Serine/threonine kinase
BCR: B-cell receptor
CDK4: Cyclin-dependent kinase 4
ER: Estrogen receptor
HIF-1: Hypoxia-inducible factor 1
GnRH: Gonadotropin-releasing hormone
ErbB: Epidermal growth factor receptor.
